# Comprehensive genome and epigenome characterization of CHO cells in response to evolutionary pressures and over time

**DOI:** 10.1002/bit.25990

**Published:** 2016-04-29

**Authors:** Julia Feichtinger, Inmaculada Hernández, Christoph Fischer, Michael Hanscho, Norbert Auer, Matthias Hackl, Vaibhav Jadhav, Martina Baumann, Peter M. Krempl, Christian Schmidl, Matthias Farlik, Michael Schuster, Angelika Merkel, Andreas Sommer, Simon Heath, Daniel Rico, Christoph Bock, Gerhard G. Thallinger, Nicole Borth

**Affiliations:** ^1^ Austrian Center of Industrial BiotechnologyMuthgasse 11Vienna1190Austria; ^2^ Graz University of TechnologyGrazAustria; ^3^ University of Natural Resources and Life Sciences ViennaMuthgasse 18Vienna1190Austria; ^4^ CeMM Research Center for Molecular MedicineAustrian Academy of SciencesViennaAustria; ^5^ Center for Genomic Regulation (CRG)BarcelonaSpain; ^6^ Research Institute of Molecular PathologyViennaAustria; ^7^ Spanish National Cancer Research Center (CNIO)MadridSpain

**Keywords:** Chinese hamster ovary cells, epigenome, genome variation, DNA‐methylation, histone modifications

## Abstract

The most striking characteristic of CHO cells is their adaptability, which enables efficient production of proteins as well as growth under a variety of culture conditions, but also results in genomic and phenotypic instability. To investigate the relative contribution of genomic and epigenetic modifications towards phenotype evolution, comprehensive genome and epigenome data are presented for six related CHO cell lines, both in response to perturbations (different culture conditions and media as well as selection of a specific phenotype with increased transient productivity) and in steady state (prolonged time in culture under constant conditions). Clear transitions were observed in DNA‐methylation patterns upon each perturbation, while few changes occurred over time under constant conditions. Only minor DNA‐methylation changes were observed between exponential and stationary growth phase; however, throughout a batch culture the histone modification pattern underwent continuous adaptation. Variation in genome sequence between the six cell lines on the level of SNPs, InDels, and structural variants is high, both upon perturbation and under constant conditions over time. The here presented comprehensive resource may open the door to improved control and manipulation of gene expression during industrial bioprocesses based on epigenetic mechanisms. Biotechnol. Bioeng. 2016;113: 2241–2253. © 2016 The Authors. *Biotechnology and Bioengineering* Published by Wiley Periodicals, Inc.

## Introduction

The epigenetic regulation and genomic variation which define the behavior of cells, for instance during development of cancer, embryogenesis, or the reprogramming of stem cells, were the focus of numerous recent studies. However, while rapid changes occur in epigenome and transcriptome upon adaptation of primary cells to in‐vitro culture, little data is available on the contribution of the above towards adaptation of continuous cell lines that may be maintained in culture under a variety of conditions or towards specific selected phenotypes.

Epigenetic mechanisms may influence gene expression both on a short term (as within a batch culture during changing environmental conditions) (Hernandez‐Bort et al., 2012; Le et al., 2013; Wippermann et al., 2014) and on a long‐term basis (as during prolonged culture periods over months or during permanent adaptation to different media/culture conditions). The later could explain the phenomenon of phenotypic drift that has been observed, for instance, in long‐term cultures (Bailey et al., 2012). Epigenetic control is conveyed via two primary, interacting mechanisms, namely DNA‐methylation, and modifications of histones. While the former tends to be more long term, histone modifications, which can have both repressing and enhancing effects on transcription, can change faster in response to environmental stimuli. The effect of these modifications is a change in chromatin structure, influencing the activity of the transcriptional machinery at the respective locus (Cedar and Bergman, 2009; Rose and Klose, 2014). This can be further modified by additional mechanisms, such as the interaction with long non‐coding RNAs or by structural DNA sequences such as matrix associated regions or ubiquitous chromatin opening elements that lead to chromatin remodeling (Brinkman et al., 2012; Sarkies and Sale, 2012). So far, these mechanisms were mostly investigated in the context of cancer and developmental biology, so that very little information is currently available on changes in epigenetic regulation in cells maintained in culture (Nestor et al., 2015; Wippermann et al., 2015). The concept of changing the epigenome globally, however, has been used to advantage, both for cell line optimisation (Seth et al., 2006) and for short‐term transcriptome modification to increase recombinant productivity by histone deacetylase inhibitors such as sodium butyrate (Kantardjieff et al., 2010; Lee et al., 2014; Liu et al., 2014). The few available studies of epigenetics in Chinese Hamster Ovary (CHO) focused on biotechnologically important issues, such as the silencing of the product gene (Osterlehner et al., 2011; Spencer et al., 2015; Yang et al., 2010), but did not investigate the global dynamics of epigenetics. Several reports indicate changes of the transcriptome during the changing nutrient and metabolite concentrations encountered by cells during batch or fedbatch culture (Hernandez‐Bort et al., 2012; Le et al., 2013), while most of the available literature compares gene expression patterns in different CHO clones that produce recombinant protein(s), trying to capture the differences that define their performance in industrial processes, with a focus on high productivity and growth (Charaniya et al., 2009; Clarke et al., 2011; Dinnis et al., 2006; Doolan et al., 2008; Nissom et al., 2006; Vishwanathan et al., 2014). Although epigenetic regulation of gene expression was proposed as one possible contributor to the diversity observed in phenotypes (Dahodwala and Sharfstein, 2014), the issue was marred by the fact that a large number of genomic variants are frequently found in continuous cell lines, due to the high number of divisions they incur (Lin et al., 2014; Weissbein et al., 2014). Similarly, continuous cell lines exhibit variation in the number of chromosomes per cell even within a clonal population, a phenomenon that occurs frequently in cancerous cell lines (Jefford and Irminger‐Finger, 2006) and is well documented for CHO cells (Derouazi et al., 2006). In addition to chromosome number aberrations, frequent translocations within and between chromosomes occur resulting in interesting and varying karyotypes (Cao et al., 2012). In addition, there are indications that this phenomenon is not entirely due to specific properties of immortal or cancer cell lines, but may be a result of in vitro culture conditions and the frequent number of divisions that cells undergo in culture, as similar observations were made in stem cells after prolonged in vitro culture periods (Weissbein et al., 2014).

We here present a study of genomic and epigenetic variations in related cell lines that underwent different evolutionary pressures, including adaptation to different media formulations, adaptation to growth in suspension, prolonged culture times and selection of phenotypic variants by cell sorting and subcloning. We use CHO cells because (i) extensive data on their genome (Brinkrolf et al., 2013; Hammond et al., 2012; Lewis et al., 2013; Schroeder Kaas et al., 2015; Xu et al., 2011), transcriptome (Hernandez‐Bort et al., 2012; Kildegaard et al., 2013; Le et al., 2013), their stability or rather instability (Barnes et al., 2003; Kim et al., 2011) as well as the diversity of phenotypes (Pilbrough et al., 2009) for this cell line is available; (ii) they are a frequently used model system for many applications, so that understanding the processes underlying adaptation and environmental response may have implications for interpretation of research results and finally; and (iii) because their widespread use as a mammalian cell factory for production of biotherapeutic proteins such as human antibodies has large economical and medical consequences (Walsh, 2014).

Both full genome sequences and DNA‐methylation patterns were obtained. In addition, short‐term response to environmental changes was analysed during a batch culture on the level of DNA‐methylation and histone modifications. All data were collated into an online resource.

## Materials and Methods

### Cell Lines and Culture Conditions

All cell lines analyzed were derived from serum‐dependent CHO‐K1 cells (ECACC CCL‐61) which were grown in 1:1 DMEM/Ham's F12 (Biochrom) containing 2% fetal calf serum (PAA) and 4 mM L‐Gln (Sigma–Aldrich), in T‐flasks at 37°C in an atmosphere containing 7% CO_2_ (FCS sample)._._ Cells were adapted to protein‐free medium and suspension growth by passaging at 1:3 dilution into CD‐CHO (Gibco/Life Technologies) with 8 mM L‐Gln and Anti‐Clumping Agent (Gibco/Life Technologies). Cells recovered within 3 weeks and were expanded into a master cell bank (MCB). All suspension‐adapted cells were grown in shaker flasks at constant shaking at 140 rpm. Cells were thawed from the MCB and allowed to recover for 2 weeks (PF‐MCB sample). Cells were then maintained in culture by passaging twice weekly for 6 months (PF‐6 months). PF‐MCB cells previously adapted to grow in the same medium without glutamine (Hernandez‐Bort et al., 2010) (no‐Gln) were sampled likewise 2 weeks after thawing as were cells from a subclone obtained by cell sorting for increased transient productivity (Pichler et al., 2011) (K1/1D9‐MCB). These high‐qP cells were maintained in culture, their transient productivity tested every month until it dropped from initially 13 pg/cell/day to 4 pg/cell/day after 3 months (K1/1D9‐3 months). Testing for transient productivity was performed by nucleofection using 20 ng plasmid DNA per 10^7^ cells as described previously (Pichler et al., 2011). All samples were taken on day 3 of an exponentially growing culture. For PF‐MCB an additional sample was taken during stationary phase (day 8) for bisulfite sequencing (see Fig. S1 for batch sampling points).

### Isolation of Genomic DNA

Genomic DNA was isolated from 5 × 10^6^ cells of a mid‐exponential culture (day 3 after seeding) following the protocol of the DNeasy Blood & Tissue Kit (Qiagen Cat. No 69504). The sample was diluted to 40 ng/μL in a total volume of 100 μL into 1.5 mL TPX microtubes (Diagenode) and sonicated in a Bioruptor (Diagenode) at 4°C using nine cycles at high setting [30 s “ON”/30 s “OFF”]. The average size obtained was verified to be 400 bp by Bioanalyzer (Agilent DNA 7500 kit).

### Library Preparation and Sequencing

Library preparation for genome sequencing was performed using KAPA Library Preparation Kit Illumina series (KK8201) following the manufacturer's guidelines. The first sample sequenced (PF‐MCB) was analyzed using an Illumina HiSeq 2000 (paired‐end, 100 bp read length), all other samples were analyzed using the upgraded Illumina HiSeq 2500 (paired‐end, 150 bp read length). Library preparation and sequencing for bisulfite‐treated samples was performed as previously described (Tomazou et al., 2015).

### Data Preprocessing for Genome and Bisulfite Sequencing

Raw reads were preprocessed with cutadapt v1.7 (adapter trimming) (Martin, 2011) using a maximum allowed error rate of 0.05 and a minimum overlap length of 4 as well as with CLC Assembly Cell clc‐quality‐trim v4.2 (quality trimming) (http://www.clcbio.com/products/clc-assembly-cell/) with a quality cut‐off of 20. The quality was assessed with FastQC v0.10.1 (http://www.bioinformatics.babraham.ac.uk/projects/fastqc/).

### Genomic Mapping and Normalization

Preprocessed reads were aligned in paired‐end mode to the Chinese Hamster reference sequence published by Brinkrolf et al. (2013) with BWA mem (Li and Durbin, 2009) using the default parameters with ‐M and ‐R flags as recommended for SNP/InDel calling in the corresponding variant calling documentation. Aligned reads were coordinate sorted with Picard SortSam v1.112 (http://broadinstitute.github.io/picard/) and indexed with SAMtools index v0.1.19 (Li et al., 2009). Duplicates were removed with Picard MarkDuplicates v1.112. The quality of the mappings was assessed with QualiMap v2.0 (García‐Alcalde et al., 2012). The mappings of all cell lines were normalized to match the coverage of the cell line with the lowest average coverage using SAMtools view v0.1.19. The average coverage was computed with QualiMap v2.0 and BEDtools genomeCoverageBed v2.17.0 (Quinlan and Hall, 2010). To normalize the PF‐MCB data with a read length of 100 bp (all other cell line data have a read length of 150 bp), the data was subsampled to the lowest average coverage for SNP/InDel analysis and to the lowest read count for SV analysis, as this analysis is based on broken read pairs.

### SNP/InDel Calling

Local realignment, SNP/InDel detection and filtering were performed using the Genome Analysis Toolkit (GATK) v2.7–4 (McKenna et al., 2010) as recommended by the GATK documentation, with default settings unless stated otherwise. The pipeline was applied on every normalized and full‐coverage dataset for each cell line separately. The GATK HaplotypeCaller was run with a minimum phred‐scaled confidence threshold at which variants should be emitted of 10 and at which variants should be called of 30 as well as with a minimum pruning of 10. Only SNP/InDels with a quality score equal or higher than 30 were included in further analyses. Summary statistics were computed using VCFtools vcf‐stats v0.1.11 (Danecek et al., 2011). SNP/InDel effects on coding sequences were evaluated using CooVar v0.07 (Vergara et al., 2012).

### Structural Variant (SV) Calling

Delly v0.5.9 (Rausch et al., 2012) was applied to call SVs with an insert size cut‐off of three (for deletions only) and a minimum paired‐end mapping quality of 20. All variants with a minimum of five broken read pairs supporting the variant as well as with a minimum length of 300 bp (for deletions, inversions, and duplications) were included in further analyses as recommended by the Delly documentation.

### Bisulfite Mapping and Methylation Calling

Preprocessed reads were aligned in paired‐end mode to the reference sequence (Brinkrolf et al., 2013) using Bowtie 2 v2.2.2 (Langmead and Salzberg, 2012) under the control of the Bismark v0.13.0 (Krueger and Andrews, 2011) pipeline (non‐default parameters: *N* = 1, *D* = 20, *R* = 3, score‐min = “L,0,−0.3”). Bismark was also used to remove duplicate reads. The quality of the mappings was assessed with QualiMap v2.0.

Methylation profiles were generated for all C contexts using Bismark v0.14.3 with default settings. Based on M‐bias plots, the first 15 bp from the 5′ end and 3 bp from the 3′ end were ignored in both reads of a pair for all samples to avoid biases in methylation calling.

### Annotation

Annotation files were downloaded from NCBI for the reference genome. SNPs/InDels were annotated by means of VCFtools vcf‐annotate v0.1.11, whereas SVs were annotated by means of Delly io‐ver v0.5.9. Methylation profiles were annotated using BEDtools window v.2.17.

### Comparative Analyses and Evaluations

The GATK SelectVariants v2.7–4 was used to compute concordance and discordance SNP/InDel lists between all cell line datasets. Comparative summary statistics were computed using VCFtools vcf‐compare v0.1.11. To compare SV variant lists between all cell line datasets, BEDtools intersect v2.17.0 with a reciprocal fraction overlap of 0.8, and a breakpoint deviation of 500 bp (for translocation only) was used, generating concordance and discordance SV lists. Finally, the supporting read counts for SNP/InDel and SV calls as well as the homozygosity (0/0) or heterozygosity (0/1) of these calls were extracted from the variant calling results (VCF files).

The differential analysis of methylation profiles was conducted using the R package methylKit v0.9.2 (Akalin et al., 2012). After coverage normalization, methylation profiles were tiled with a window size of 1 kb (step size 1 kb) and filtered to keep only regions covered in all samples. DMRs were called with a minimum difference in methylation of 25%, a minimal coverage threshold of three and a *Q*‐value < 0.1 (based on SLIM [Wang et al., 2011]) using Fisher's exact test. R (R Core Team, 2013) and the Bioconductor package GenomicRanges (Lawrence et al., 2013) were used for assignment of called DMRs to genomic features and preparation of summary statistics and plots.

### Impact of Coverage on Identification of Variants

To estimate the effect of coverage on the reliability with which variants are called unique at a coverage of 13.5, the reads of K1/1D9‐MCB, available at 21.5× coverage, were subsampled to three sets of 13.5× each, which were compared against each other both for SMs and SVs. The reads of PF‐MCB, available at 37.5×, were likewise subsampled to 20× and compared. In addition, K1/1D9‐MCB and K1/1D9‐3 months, both available at 21× coverage, were compared at this coverage in addition to the standard 13.5x× (Suppl. File‐WS13).

### Batch Culture for ChIP‐Seq—Cell Fixation and Immunoprecipitation

PF‐MCB cells were seeded into eight parallel 500 mL shaker flasks at 2 × 10^5^ cells/mL, with working volumes of 250 mL. The cultures were analyzed twice daily for total and viable cell count using a ViCell analyzer (Beckmann Coulter). Samples were taken as indicated in Suppl. File‐WS14 from each flask and pooled for ChIP‐seq analysis.

Cell fixation and subsequent lysis of 10^7^ cells were performed following the protocol of the Shearing ChIP kit by Diagenode (kch‐redmod‐400). Sonication was performed by Bioruptor (Diagenode) using 1 run of 10 cycles [30 s “ON”, 30 s “OFF”] at high power setting, 10 min on ice, vortex and spin down, 1 run of 10 cycles [30 s “ON”, 30 s “OFF”] at high power setting, 10 min on ice, vortex and spin down, 1 run of 15 cycles [30 s “ON”, 30 s “OFF”] at high power setting, 10 min on ice, vortex and spin down, and 1 run of 5 cycles [30 s “ON”, 30 s “OFF”] at high power setting. At this point sheared chromatin was stored at −80°C after snap freezing in liquid Nitrogen. Magnetic immunoprecipitation was performed following the protocol from the iDeal ChIP‐seq kit by Diagenode (AB‐001‐0024) using 10^6^ cells per 2 μg antibody (H3K4me1, H3K4me3, H3K9me3, H3K27ac, H3K27me3, H3K36me3, and IgG as negative control). DNA concentration was quantified using Quant‐IT Picogreen dsDNA (N°P7589, Life Technologies). DNA quality control and recovery were assessed by Real‐time PCR using TSS‐GAPDH (Forward‐primer: CCCTTGAGCTGTGACTGGAT, Reverse‐primer: CACTCTGCGGTTTTCACCTG) as positive control target and target myoglobin exon 2 as provided by the kit as negative control. Final control of size and quantity was performed on a Bioanalyzer (Agilent DNA 7500 and DNA 12000 Kit—C5067‐1506).

### Data Preprocessing and Chip‐Seq Mapping

Raw reads were preprocessed with Flexbar—v2.4 (Dodt et al., 2012) using a minimum overlap of adapter and read sequence of 6, 2 allowed mismatches, and gaps per 10 bases overlap and 18 as a minimum read length to remain after removal. The quality was assessed with FastQC v0.10.1 (http://www.bioinformatics.babraham.ac.uk/projects/fastqc/). Although the required depth depends on the nature of the mark and the state of the cells in each experiment, ChIPseq experiments in mouse (Jung et al., 2014) suggest that a depth of 25 million reads are sufficient. Therefore only those samples with more than 25 million reads that passed the filter (50 bp read length) were used. Preprocessed reads were aligned in single end mode to the Chinese Hamster reference sequence published by Brinkrolf et al. (2013) with BWA mem v0.7.7 with default parameters. Aligned SAM files were converted to BAM files with SAMtools view v0.0.18. Aligned BAM files were coordinate sorted with Picard SortSam v1.95 and duplicates removed with Picard MarkDuplicates v1.95 (http://broadinstitute.github.io/picard/).

### Chip‐Seq Peak Calling and Genome Annotation

The fragment size was modeled using the PhantomPeakQualTools (http://code.google.com/p/phantompeakqualtools) R script. MACSv2.0.10 (Zhang et al., 2008) peak caller was used to compare ChIP‐seq mapped reads to a corresponding input sample sequenced control with the fragment size predicted by PhantomPeakQualTools. Both the standard method for H3K4me3 and H3K27ac and the—broad flag method for H3K4me1, H3K27me3, H3K36me3, H3K4me9 with default parameters were used. All samples were normalized by sequencing depth in million reads with the flag—SPMR. ChIPseeker package (Yu et al., 2015) and the Genomic Features package for R (v.3.02) (Lawrence et al., 2013) were used to annotate the location of normalized peaks relative to genomic features.

### Chromatin State Learning Across Time Points

Chromatin states were learned by applying the ChromHMM hidden Markov Model v1.10 (Ernst and Kellis, 2012) at 200 bp resolution to the six histone marks (H3K4me3, H3K4me1, H3K9me3, H3K27ac, H3K27me3, H3K36me3) for each time point. ChIP‐seq mapped reads were converted to BED files with BEDTools v2.16.2. Data was binarized in 200 bp using the BinarizeBed function of ChromHMMv1.10, using the input sample sequenced as a control. After testing a range of possible states, an 11 state model was trained with the full data set, to identify biologically meaningful patterns similar to previously published states from human cell types (Roadmap Epigenomics Consortium, 2015) using the ChromHMM LearnModel function. Each time point was segmented using the 11 state model by the MakeSegmentation function, resulting in a segmentation covering 99.8% of the genome. The enrichment of each state of the segmentation was computed for a set of external coordinates (CpG islands, mRNA, exon, TSS, transcription end site (TES) and regions within 2 kb of the TSS) in BED format. Finally, the enrichment of each state at fixed positions relative to anchor positions was computed with the NeighborhoodEnrichment function. Chromatin state coverage was plotted for all reads with ggplot package for R (v.3.02).

### Relationship Between Histone Marks and Methylation

An intersection between chromatin states and CpG‐methylation coordinates with a minimum read Coverage of three was calculated with BEDTools v2.16.2 and a boxplot generated with ggplot2 package for R (v.3.02).

### Differential Binding Analysis ChIP Seq Data

Peaks were subjected to DiffBind (http://bioconductor.org/packages/release/bio/DiffBind/inst/doc/DiffBind.pdf) analysis to perform differential analysis of histone modifications. The binding matrix was calculated with affinity scores based on TMM normalization (EdgeR), using read counts minus control read counts and Effective Library size. PCA plots were generating using affinity data for all sites. Then, a contrast between the three culture phases was established to run edgeR analysis to identify differentially bound sites using the default threshold of FDR > = 0.1.

## Results

**Figure 1:**
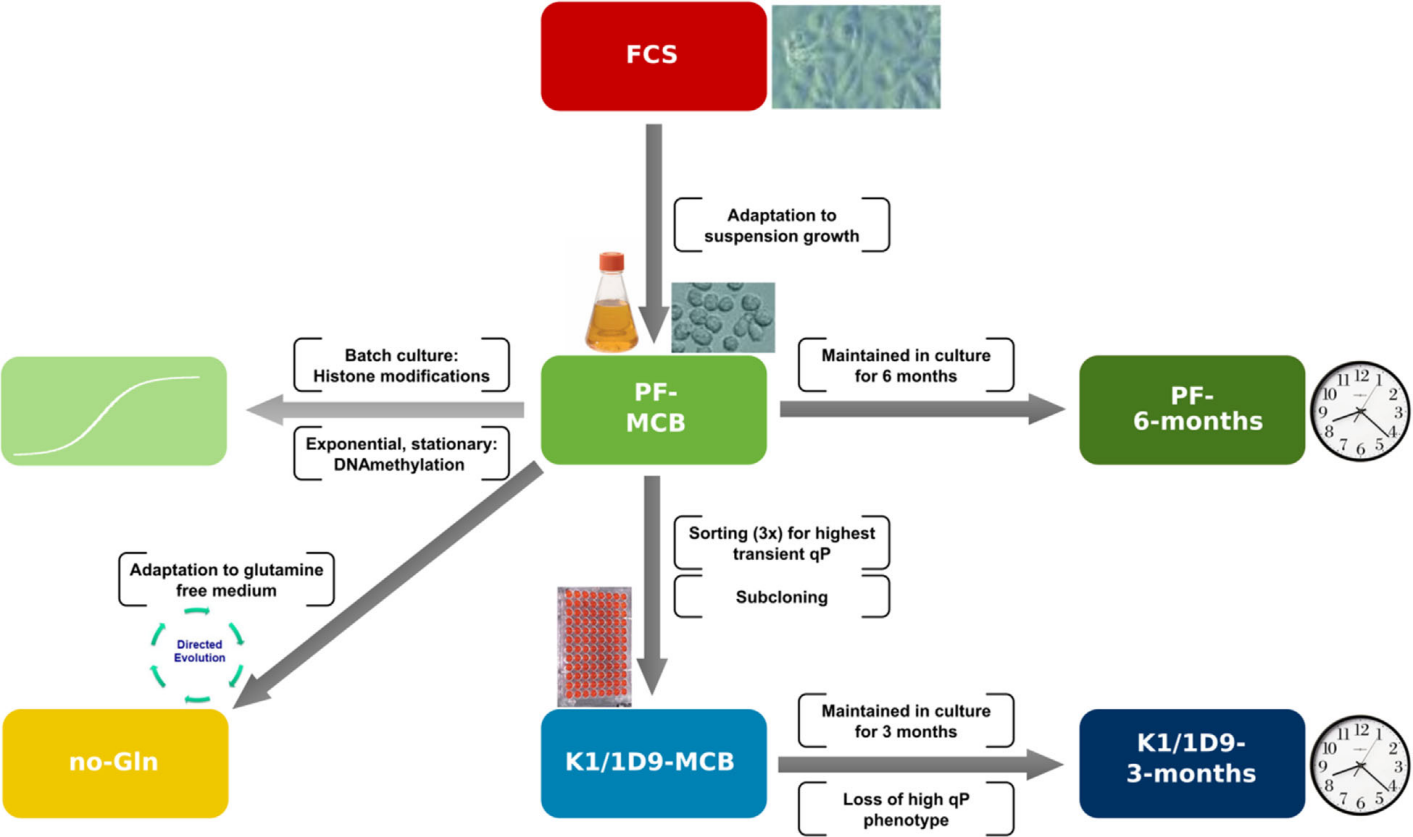
Cell lines analyzed, their relationship and development. K1‐ECACC cells grown adherent in FCS (FCS) were adapted to protein free suspension growth (PF‐MCB) and subsequently cultivated for 6 months (PF‐6 months). PF‐MCB cells were adapted to growth in glutamine free medium (no‐Gln). A subclone isolated for increased transient productivity was isolated by repeated cell sorting and subcloning (K1/1D9‐MCB). This subclone was also passaged for 3 months and reanalyzed (K1/1D9‐3 months). Colors shown are used in all figures.

For our study, six related cell lines (Fig. 1) were sequenced both for their genome sequence and DNA‐methylation pattern. The parental cell line CHO‐K1 grown adherent in FCS supplemented medium (FCS) was adapted to growth in suspension in a protein‐free medium, containing 8 mM glutamine, and a master cell bank established (Pf‐MCB). This cell line was also used for analysis of histone modifications and changes in DNA‐methylation patterns during batch culture. After the initial sampling, cells were passaged for 6 months and reanalyzed (Pf‐6 months). Pf‐MCB cells were adapted to grow at similar growth rate and to high cell density in the same medium lacking glutamine, the main energy source in vitro, which required metabolic modifications to ensure sufficient energy supply (no‐Gln) (Hernandez‐Bort et al., 2010). In addition, a subclone isolated for threefold higher transient productivity (Pichler et al., 2011) was analyzed at two time points, 2 weeks after thawing (K1/1D9‐MCB) and 3 months later, after the phenotype of high productivity was lost (K1/1D9‐3 months). Importantly, except for K1/1D9, cells were adapted to the new culture conditions without subcloning, so represent pools. To enable a valid comparison, all genomes were subsampled to the lowest coverage (13.5‐fold) for analysis of SNPs and InDels <5 bp (from now on summarized as small mutations or SM) (Suppl. File WS2). For structural variant (SV) comparison, subsampling was done to the matching number of reads rather than coverage, as analysis for SVs is based on the number of broken read pairs. All reads were mapped against the chromosome assigned Chinese Hamster draft genome (Brinkrolf et al., 2013).

### Genomic Variants Occurring During Cell Line Evolution

**Figure 2:**
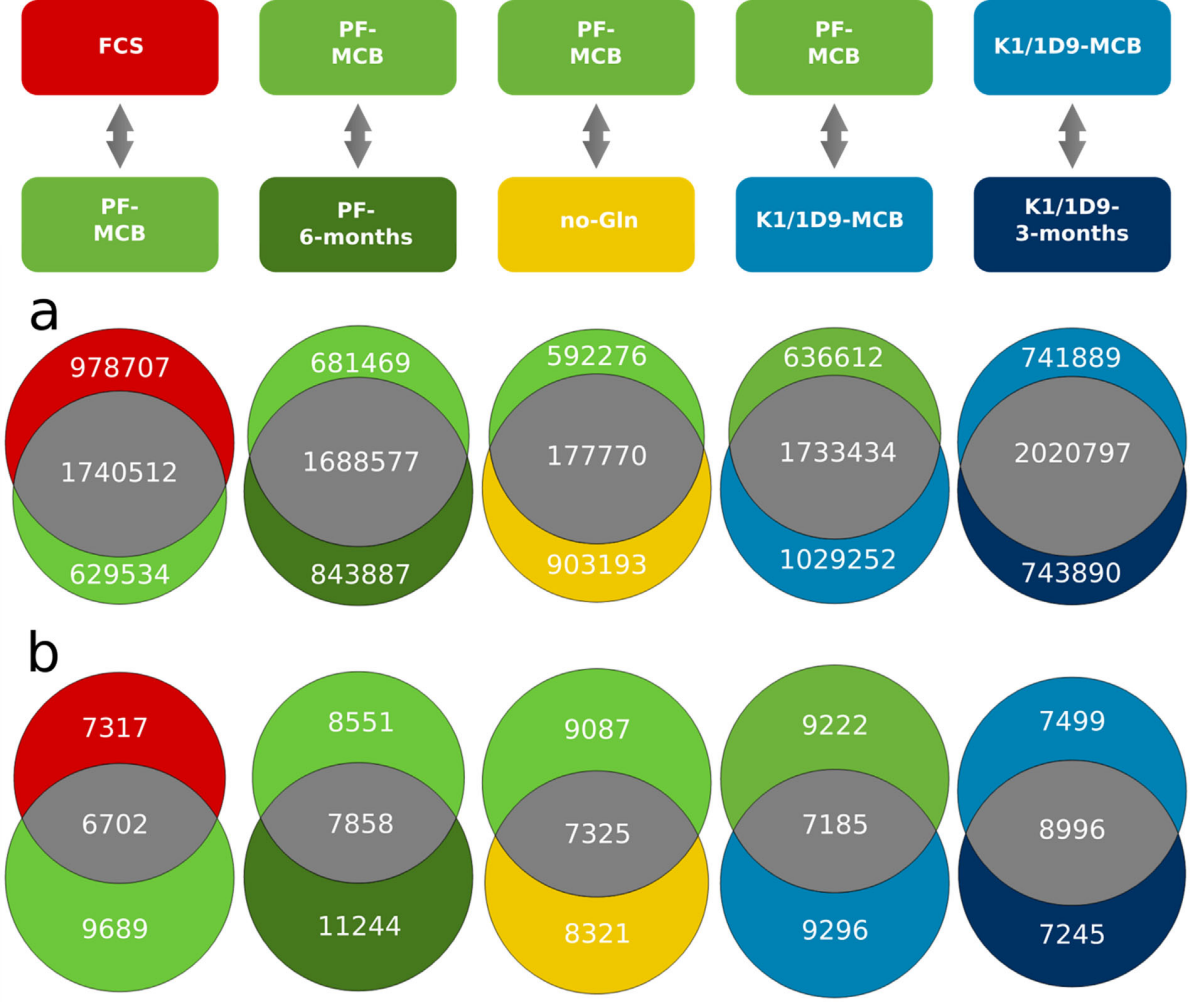
Comparative analysis of changes in SMs (**a**) and SVs (**b**). Venn diagrams are size proportional for each comparison, but not across all cell lines. The variants unique for each cell line are presented in the respective colour assigned to the cell lines.

The first question investigated was the occurrence of genomic modifications during adaptation and selection and over time in culture. In Figure 2a and b, the shared and cell line specific calls of SMs and SVs are presented for comparisons of each cell line relative to the next stage of its evolution (absolute numbers and percentages are listed in Suppl. File WS5 and WS9). Comparative analysis of SMs indicates that 51–57% of these are shared between the two cell lines compared in each case and the rest are uniquely found in one or the other cell type, with SMs both disappearing and new ones appearing at each transition. We observe a larger percentage of SMs lost than gained relative to the total number of SMs within that comparison during the transition from adherent culture to protein‐free suspension culture (−29% vs. +19% for FCS vs. Pf‐MCB), which suggests the selection of a suspension compatible genotype. Subsequently, during selection of specific phenotypes such as growth in glutamine‐free medium or enhanced transient productivity, more SMs are gained than lost (−18% vs. +28% for Pf‐MCB vs. no‐Gln and −19% vs. +30% for Pf‐MCB vs. K1/1D9‐MCB, respectively). Overall, only ∼1.0–1.2% of SMs have a direct effect on protein sequence, while 98.5% are intergenic or intronic (Suppl. File WS4).

The variation in SVs, consisting of duplications, deletions, inversions, and translocations, is much higher, with only approx. 30% shared in each comparison. In all cell lines, the majority of SVs consist of translocations (80% or more). Similar to the SMs, the total number of SVs found in each population is comparable, with the highest absolute number found in PF‐6 months. The higher overlap in SVs between K1/1D9‐MCB and K1/1D9‐3 months (37.9% shared SVs vs. 28.4% in the comparison of PF‐MCB against PF‐6 months) may be explained by the shorter period that passed between the two sampling points. A simulation using subsampled datasets derived from a single sample available at higher coverage also results in unique calls for each subsample (Suppl. File WS13), an effect that decreases with higher coverage. Nevertheless, even at 13.5× coverage, the percentage of unique calls in the two‐sample comparison (1D9‐MCB vs. 1D9‐3 months) is distinctly higher than in the subsample comparison from the same population (1D9‐MCB vs. MCB subsamples), thus supporting the theory of continuously occurring rearrangements in the CHO genome.

**Figure 3:**
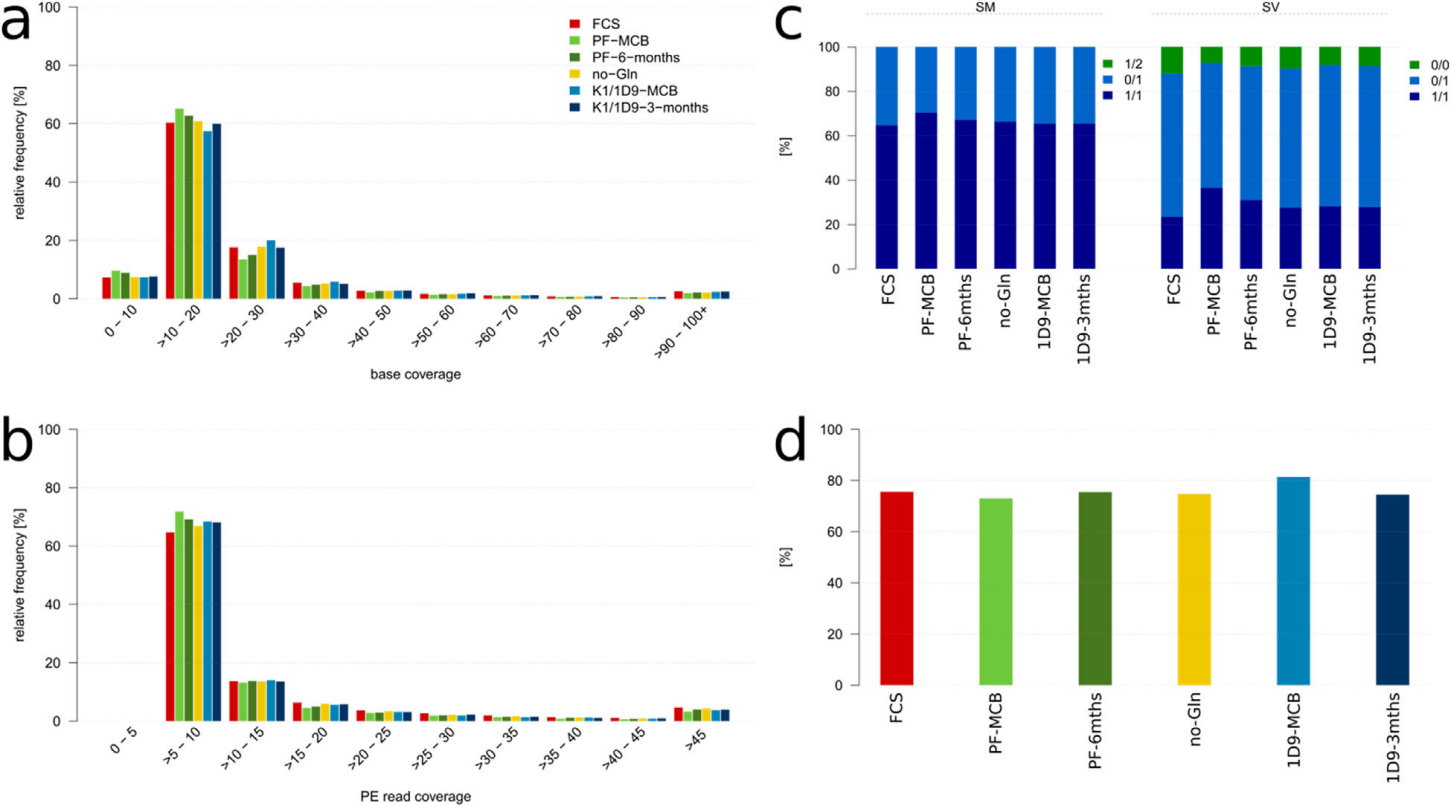
Distribution of supporting read depth for SM and SV calls across all cell lines and subclones derived from the variant calling results. **a**: Number of reads supporting SMs (base coverage). **b**: Number of broken paired‐end reads supporting SVs (PE read coverage) **c**: Homozygosity/heterozygosity of SMs (left) and SVs (right). The genotype for diploid calls is encoded as allele values in the format 0/0, 0/1, 1/1, and 1/2. The allele values are 0 for the reference allele, one for the most frequent alternative allele and two for the second most frequent alternative allele. The exception is 0/0 variants for SVs, which are rare variants indicative of high diversity. Not visible in the figure are 1/2 calls for SMs (two different SNPs at the same position: less than 0.1% in all cell lines). **d**: Percentage of translocation calls below quality threshold but emitted by the caller.

An important question in this context is whether, on the genome level, clones are more homogenous than pools. Variant calling in populations comprising several subpopulations with more widely varying genotypes (as expected in a pool) would lead to (i) a higher number of variants with lower alternative allele frequencies and confidence levels (Fig. 3a and b); (ii) a generally higher number of variant calls with a larger proportion of heterozygous variants (Fig. 3c); and/or (iii) with confidence levels below the threshold considered significant for a given coverage (Fig. 3d). The distribution of homozygosity and heterozygosity for SVs is more diverse than for SMs, with a larger number of heterozygous SVs (0/1) and a significant fraction of variants with a small alternative allele frequency (0/0). In all cases, however, there is no significant difference in distribution between the pools and the clonal cell lines. In addition, the percentage of translocations identified below the quality threshold ranges from 73–82%, with the highest percentage actually found in subclone K1/1D9‐MCB. Thus, on the level of genome homogeneity, the diversity of a pool of cells is comparable to that of this clone derived from a continuous, immortalized cell line.

### DNA‐Methylation Changes During Cell Line Evolution

**Figure 4:**
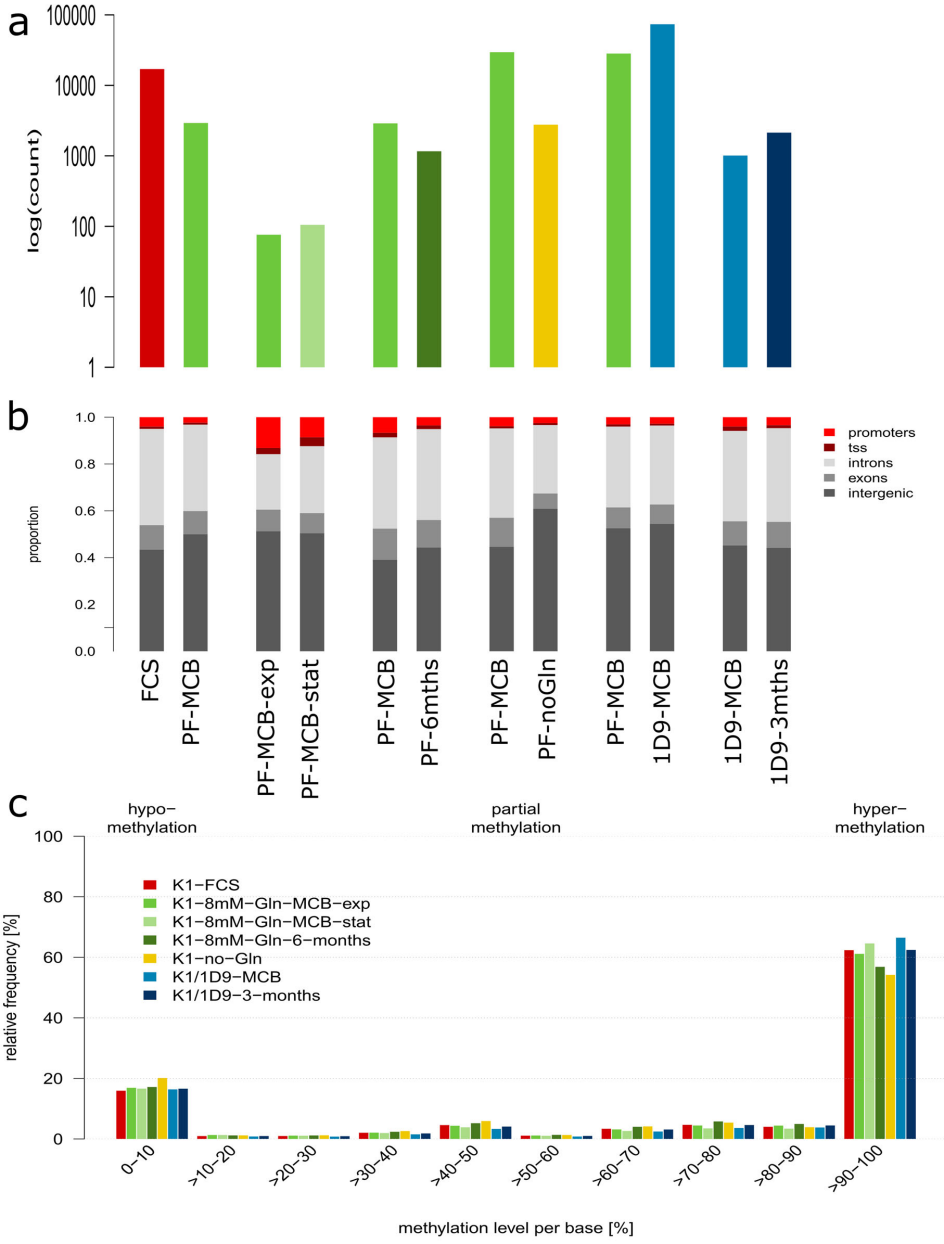
DNA‐methylation in CHO cell lines. **a**: Number of hypermethylated regions of 1 kb in each comparison. **b**: Functional annotation of hypermethylated regions in each comparison. **c**: Distribution of degree of methylation per CpG.

Bisulfite‐treated full genome DNA‐sequences were mapped against the reference genome (Brinkrolf et al., 2013) and differentially methylated regions of 1 kb (DMR) assigned to promoter regions, transcription start sites (TSS), introns, exons, and intergenic regions (Fig. 4a and b, Suppl. File WS11). Noticeably, time in culture has the least effect on differential methylation (total number of DMRs: 2,947 for PF over 6 months and 2,480 for K1/1D9 over 3 months), indicating that DNA‐methylation patterns are largely passed on to daughter cells under constant culture conditions. In contrast, adaptation to different media/growth conditions causes intermediate changes in DMRs (13,674 DMRs for transition from FCS‐containing to protein‐free medium and 21,788 DMRs for adaptation to glutamine‐free medium). The highest number of DMRs (69,040) is found in the comparison of the K1/1D9‐MCB subclone with the parental PF‐MCB, where its establishment involved several rounds of sorting for this specific phenotype (top 1% productivity) and a final round of subcloning (Pichler et al., 2011). Overall, 55–65% of all CpGs are fully methylated, and 15–20% fully demethylated (Fig. 4c). Hypermethylation of CpGs is highest in K1/1D9‐MCB and lowest in no‐Gln, and it decreases over time in culture both for K1/1D9 and PF. Summarized, these results indicate that transcription patterns to fit a given set of culture conditions are laid down by corresponding DNA‐methylation patterns which are inherited by daughter cells, but may be altered upon a change in culture conditions.

### Epigenetic Response During Batch Culture

**Figure 5:**
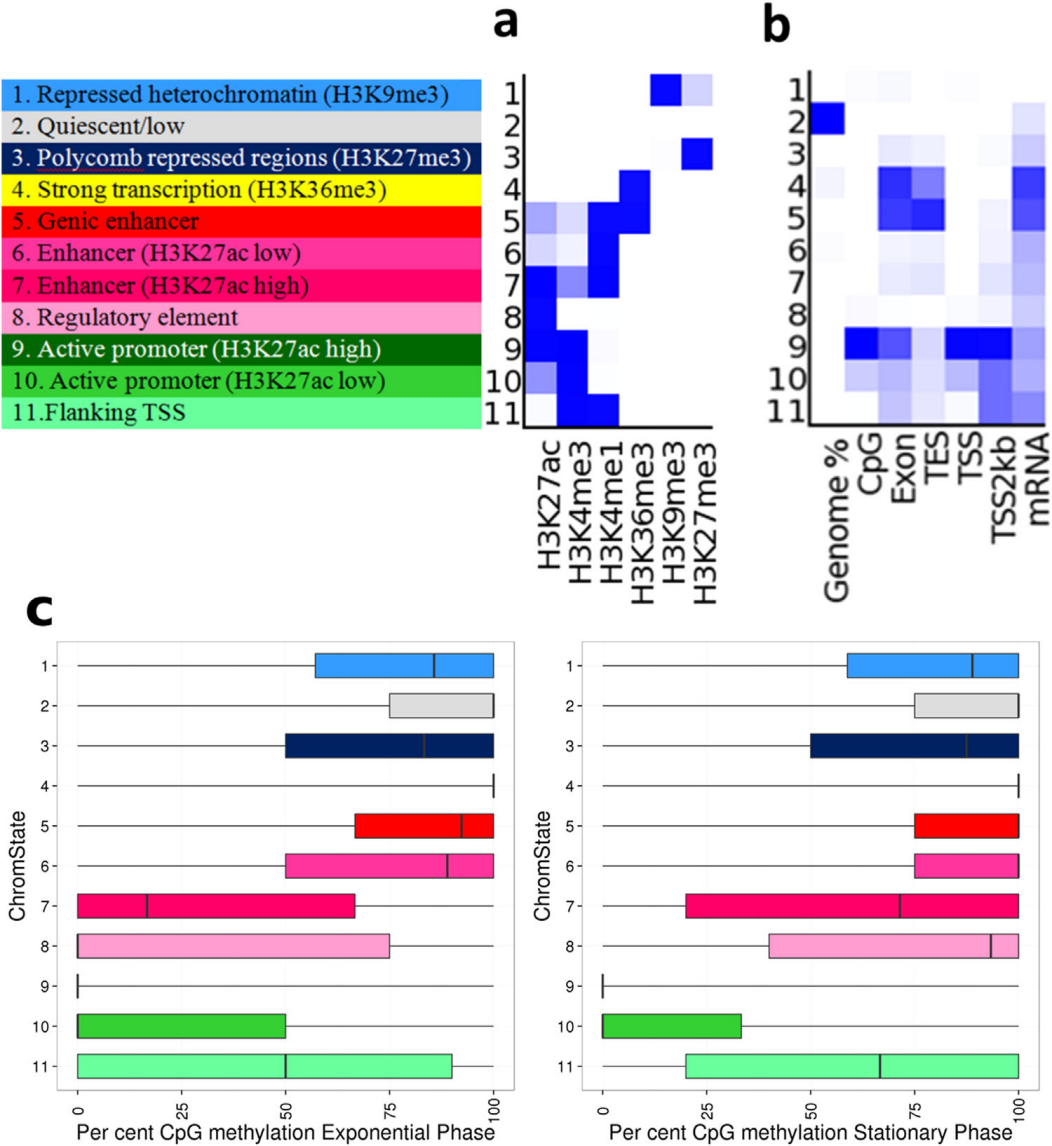
Chromatin states defined by histone modifications. Colour code and chromatin state numbers are the same as used in the JBrowser. **a**: Eleven states based on six histone modification marks. The active states consist of active transcribed states, enhancer states and promotor states. The inactive states consist of constitutive heterochromatin, repressed Polycomb states, and a quiescent/unmarked state. **b**: Enrichment of states for CpG‐islands, exon, transcript end site (TES), transcript start site (TSS), regions 2  kb upstream of the TSS, and mRNA for the 66 h sample. **c**: Relative DNA‐methylation of CpGs in the 11 chromatin states during exponential and stationary growth.

**Figure 6:**
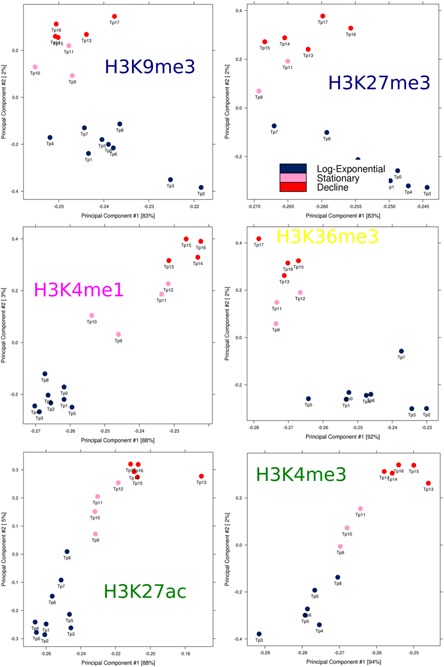
PCA analysis of histone modifications during batch culture: Timepoints (Tp) are coloured according to culture phase as indicated.

In the course of a batch culture, with its declining nutrient availability and increasing waste metabolite concentrations, cells need to respond by rapidly changing gene transcription. PF‐MCB cells were seeded into shaker flasks and sampled every 12 h for ChIP‐seq analysis of six histone modifications as recommended by the International Human Epigenetics Consortium (http://ihec-epigenomes.org/research/reference-epigenome-standards/) (Suppl. File WS14). Using ChromHMM (Ernst and Kellis, 2012), a model consisting of 11 defined chromatin states corresponding to putative promoters, enhancers, transcribed or heterochromatic regions was generated and these states assigned to in total 11,642,738 segments of 200 bp of the genome (Fig. 5). The majority of the genome (∼89%) is either quiescent (very low or no signal) or repressed (Suppl. File WS13). Overall, ∼22,3% of the 200 bp segments change state during the course of the batch culture. Comparing DNA‐methylation patterns in exponential (day 3) and stationary (day 8) phase, 143 regions of 1 kb are differentially methylated, of which only 19 are in annotated promoter regions (Suppl. File WS11). Intersection of CpG‐methylation with the ChromHMM model reveals high methylation in areas of high transcriptional activity, while highly active promoters (state 9) are fully de‐methylated (Fig. 5c). Methylation of promoters with weaker activity (state 10) range from fully de‐methylated to semi‐methylated, which might indicate activity in one allele and inactivity in the other. While there are few DMRs between these two culture stages, the percentage of methylation in the active chromatin states 4–11 do shift to higher levels of methylation, indicating that those changes in DNA‐methylation that occur are predominantly found in active regulatory regions. PCA analysis of histone modifications reveals that highly active, transcription related modifications undergo a continuous adaptation during the batch culture, while repressed regions undergo fewer changes (Fig. 6; Table I). However, for all marks there is a clear separation between these three culture phases: exponential growth (tp 0–8 = 0–101 h), stationary growth (tp 9–12 = 114–149 h) and decline phase (tp 13–17 = 162–210 h). Together, these results indicate that short‐term regulation of transcription is a continuous, adaptive process that is primarily controlled by alterations in histone modifications.

**Table I:**
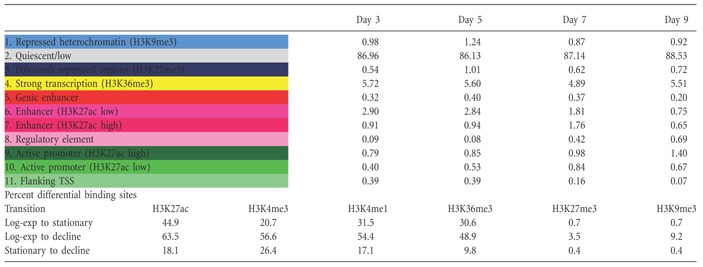
Percentage of chromatin states in the genome during batch culture and differential histone modification binding sites

## Discussion

Comparison of the different cell lines reveals that changes occur at all levels, including SMs, SVs, and DNA‐methylation, but are not necessarily linked to the process of adaptation or selection. New SMs and SVs occur also simply during time in culture. The highest degree of variation occurs with SVs, most prominently with translocations, confirming previous reports of chromosomal rearrangements and irregularities in numbers (Cao et al., 2012; Derouazi et al., 2006). Such genomic inconsistencies have also been reported in another immortalized cell line, HEK293 (Lin et al., 2014). Strikingly, variants disappear to a similar extent as new ones appear which can only in part be explained by the sequencing coverage used. Overall, the genome of immortalized cells is seemingly undergoing a continuous and random rearrangement, where certain variants may be prohibitive and therefore are immediately lost (if they impact a vital gene or functionality), while others are carried along or may even be expanded if they provide a slight growth advantage. As only a small percentage of SMs have a direct effect on protein sequence, there presumably is, for most SMs, no evolutionary pressure that promotes or removes them from the population. We note that the number of variants, their distribution and frequency within a population is not higher in a pool of cells compared to the subclone. Given the observed large number of variants that are continuously generated and lost, a “clone” would accumulate a comparable number of variants during the 50–70 population doublings required to expand it into a cell bank, thus essentially eliminating the initial genomic homogeneity of a single cell.

While genomic diversification appears to be a continuous process, changes in DNA‐methylation patterns are more significantly linked to changes in phenotype or culture conditions. Both adaptation to different media (as with most cell pools analyzed) and selection/subcloning for a specific phenotype (high transient productivity, as with subclone K1/1D9) generates prominent changes in DNA‐methylation patterns. In contrast, long‐term maintenance under constant culture conditions results only in minor changes in DNA‐methylation. Interestingly, subclone K1/1D9 has the highest overall degree of CpG‐methylation and the largest number of differentially methylated promoters relative to its parent. This confirms a recent transcriptome analysis of this cell line where two thirds of differentially expressed genes were downregulated (Harreither et al., 2015), indicating that a high productivity phenotype is only in part achieved by enhanced expression of genes needed for production, but just as importantly requires focusing on this specific task by reducing expression of unrelated functions and pathways. Finally, the lowest overall level of CpG‐methylation was observed in no‐Gln cells, which were harshly treated during their adaptation and may need to express more genes to enable survival and successful growth under such minimal conditions.

At the current state of the Chinese Hamster and CHO reference genomes, the chromatin state model will be a valuable resource to help with the annotation of promoters, TSS, regulatory regions and both coding, and non‐coding genes. Although active transcription states in many cases overlap with annotated genes, frequently transcription can be observed also in unannotated regions, while in other instances promoter regions are found in the middle of an annotated gene, possibly incorrectly assembled, although a translocation in the CHO genome cannot be excluded. The correctness of active promoter prediction is confirmed by the overlap of predicted active promoters with highly demethylated regions. While DNA‐methylation in annotated promoter regions is not notably altered during the batch culture, approx. 22.3% of the chromatin state segments of the genome undergo changes, indicating that short term changes in transcription in CHO cells are controlled by histone modifications and that these occur in a continuous adaptation to the altering conditions. Exponential growth, stationary growth, and decline phase can be clearly distinguished by PCA. The continuous adaptation during exponential growth phase is most prominent in histone marks that define regulatory units or promoters (H3K27ac, H3K4me1, and H3K4me3), thus demonstrating the efficiency of cells in achieving homeostasis in the form of constant output (= growth rate) during this phase in spite of decreasing nutrient availability, but also indicating the need for continuous adaptation.

In view of common procedures during cell line development, which typically include the requirement for subcloning, it is striking that the genome homogeneity of the analyzed subclone is not significantly different from that of the pools. Thus, the common expectation that a clone is more homogeneous should be questioned when working with fast growing, immortalized cell lines, as the divisions required to expand cells to sufficient numbers for a master cell bank or a bioreactor allows for accumulation of high numbers of new variants. At the epigenetic level, we observe that DNA‐methylation plays a major role in defining and differentiating between phenotypes and is inherited by daughter cells. Besides established approaches of changing the epigenome, such as addition of sodium butyrate or valproic acid, which both have a global impact on epigenetic regulation, other, so far unexplored mechanisms of targeted control of gene expression could be put to good use, including long‐non‐coding RNAs (Holoch and Moazed, 2015), or the application of site specific (de‐)methylases or histone modifying enzymes (Hilton et al., 2015; Lang et al., 2015). Such methods open up new options for control of cell behavior that, compared to classical overexpression or knock‐out engineering, may be easier to achieve on the one hand, but also more difficult to stabilize and maintain on the other. In either case, they would significantly enlarge the toolbox for metabolic engineering of cell behavior.

Summarized, our results suggest that culture conditions support and enforce a given phenotype as defined by the DNA‐methylome which in turn defines the gene expression pattern required for this set of conditions. Short‐term adaptation to rapidly changing nutrient availability as observed during a batch culture on the other hand is primarily controlled via a continuous modulation of histone modifications. Insights gained from these data will improve our understanding of the dynamics of the genome and epigenome in cells that are used as model systems for many basic research questions and that are maintained in culture under a variety of conditions and over prolonged periods of time. In view of the industrial use of CHO as a cell factory, they may also guide future developments towards improved epigenetic control and manipulation of cell behavior in the context of industrial bioprocesses.

## Accession Codes And Jbrowse Access

DNA‐seq and BS‐seq raw data: PRJEB9185; ChIP‐seq results: PRJEB9291. All data are available for download and visualized in a JBrowse server available at cho‐epigenome.boku.ac.at/ with a link provided from www.CHOgenome.org.

This study was supported by the Austrian Center of Industrial Biotechnology, a COMET K2 competence centre of the Austrian Research Promotion Agency FFG. Genome Sequencing was performed at the NGS Facility at Campus Science Support Facilities GmbH (CSF), member of Vienna Biocenter (VBC), Austria. Epigenome sequencing was performed at the Biomedical Sequencing Facility (BSF) at CeMM, Austria. IHL and VJ received support from FWF, the Austrian Science Fund, within the Doctoral Program “BioTechnology of Proteins” W1224, which also covered funding for open access charges.

## Supporting information

Additional supporting information may be found in the online version of this article at the publisher's web‐site.

Supporting Information.
