# Supplementary material for: Comprehensive genome and epigenome characterization of CHO cells in response to evolutionary pressures and over time
Source: Biotechnol Bioeng. 2016 Apr 29;113(10):2241–53. doi: 10.1002/bit.25990 (PMC5006888; doi:10.1002/bit.25990)

WS1: Read me

1	**Read me**	
	**Work sheet name**	**Description**
2	Mapping and Normalisation	Reads, trimming and coverage results; normalisation of sequencing for comparison
3	SM Calls	Summary of called small mutations
4	SM Annotation	Distribution of small mutations in transcripts and exons
5	SM Comparison	comparison of small mutations between cell lines
6	SNP Effect	SNP effect summary tables
7	SV Calls	Summary of called structural variants
8	SV Annotation	Distribution of structural variants in transcripts and exons
9	SV Comparison	Comparison of structural variants between cell lines
10	Methylation mapping	
11	Methylation comparison	Differential methylation of 1kb windows
12	Differential methylation	Genes with differentially methylated promoters between exponential and stationary culture
13	Subsampling	Subsampling of higher coverage datasets to 13.5x and 20x coverage to estimate erronous unique calls
14	Batch culture	sampling points and growth curves of batch cultures

WS 2: Mapping and Normalisation

Mapping and Normalisation

Overview

Name	Per base coverage w/o 0	# Raw reads	# Filtered reads	# Mapped reads	% Mapped reads	Mean insert size	Mean mapping quality	% Genome fraction for 3x coverage	% Genome fraction for 5x coverage
FCS	17,76	400.157.974	310.711.324	311.026.969	98,34	256,15	48,35	88	87
PF-MCB	37,53	1.107.865.940	954.523.903	927.162.696	96,52	260,26	47,07	89	88
PF-6months	13,51	251.429.388	229.321.924	229.635.226	97,97	341,47	47,82	87	84
no-Gln	18,45	401.219.720	318.470.571	319.287.804	98,29	284,17	47,72	88	87
K1/1D9-MCB	21,51	431.458.796	366.446.667	367.430.055	98,30	306,80	48,42	88	87
K1/1D9-3months	20,04	411.913.674	344.400.836	344.951.606	98,35	286,95	48,56	88	87

Normalisation

Name	Subsampled coverage	Subsampling type	# Mapped reads	% Mapped reads	Mean insert size	Mean mapping quality	Genome fraction for 3x covera	% Genome fraction for 5x coverage
FCS	13,52	Subsampled to coverage	236.688.425	98,34	255,70	48,33	87	84
PF-MCB-coverage	13,51	Subsampled to coverage	333.717.669	96,52	259,58	47,01	87	84
PF-MCB-reads	9,30	Subsampled to number of reads	229.601.563	96,52	258,76	46,98	84	76
PF-6months	13,51	Reference	229.635.226	97,97	341,47	47,82	87	84
no-Gln	13,51	Subsampled to coverage	233.890.453	98,29	284,43	47,70	87	84
K1/1D9-MCB	13,51	Subsampled to coverage	230.831.839	98,30	305,39	48,41	87	84
K1/1D9-3months	13,51	Subsampled to coverage	232.603.428	98,35	287,99	48,54	87	84

Notes

BWA mem v0.7.5 was used for read alignment.
Note that BWA maps reads muliple times. Therefore, the read count for mapped reads can be slightly higher than number of trimmed reads
QualiMap v2.0 was used for mapping statistics.
Normalisation was achieved by subsampling to the lowest coverage or by subsampling to the number of reads using SAMtools view v0.1.19.
Lowest coverage: 13.5 (PF-6months).
Note that the read length of the Pilot project is 100 bp and 150 bp for the rest of the strains.
Note Pilot project strain was also subsampled to number of reads of PF-6months as SV detection based on broken pairs.

Legend

Name	Mapping for a given cell line
Per base coverage	Average per base coverage of the mapping excluding 0 values
# Raw reads	Total number of raw reads
# Mapped reads	Total number of mapped reads
# Filtered reads	Total number of filtered reads
% Mapped reads	Percentage of mapped reads
Mean insert size	Mean insert size in bp
Mean mapping quality	Mean mapping quality, phred scaled score
Subsampled coverage	Subsampling coverage reached
Subsampling type	Subsampling to the lowest coverage or by subsampling to the number of reads
% Genome fraction for 3x cove	Percent of genome with at least 3fold coverage
% Genome fraction for 5x cove	Percent of genome with at least 5fold coverage

WS3: SM Calls

Normalised Small Mutations

FCS	
# Variants	2.719.219
# InDels	385.628
# SNPs	2.334.092

PF-MCB-coverage	
# Variants	2.370.046
# InDels	333.699
# SNPs	2.036.673

PF-6months	
# Variants	2.532.464
# InDels	381.366
# SNPs	2.151.496

no-Gln	
# Variants	2.680.963
# InDels	389.870
# SNPs	2.291.487

K1/1D9-MCB	
# Variants	2.762.686
# InDels	410.958
# SNPs	2.352.255

K1/1D9-3months	
# Variants	2.764.687
# InDels	405.496
# SNPs	2.359.707

Notes

Small mutations (SM) were called with the Genome Analysis Toolkit (GATK) v2.7-

Quality cutoff 30 (phred score)

Subsampled to the lowest coverage of PF-6months

Legend

Variants: total number of variants called with GATK

InDels: total number of InDels called with GATK

SNPs: total number of SNPs called with GATK

WS4: SM Annotation

Annotated Normalised Small Mutations

Name	Total # SMs	# Variants in mRNAs	% Variants in mRNAs	# Variants in exons	% Variants in exons
FCS	2719219	772057	28,39	82951	3,05
PF-MCB	2370046	713966	30,12	74546	3,15
PF-6months	2532464	737990	29,14	78386	3,10
no-Gln	2680963	790406	29,48	84983	3,17
K1/1D9-MCB	2762686	810695	29,34	89459	3,24
K1/1D9-3months	2764687	797104	28,83	87343	3,16

Notes
Small mutations (SM) were annotated with VCFtools vcf-annotate v0.1.11

Legend	
Name	Cell line name
Total # SMs	Total number of SNPs/InDels
# Variants in mRNAs	Number of SNPs/InDels in mRNAs
% Variants in mRNAs	Percentage of SNPs/InDels in mRNAs
# Variants in exons	Number of SNPs/InDels in exons
% Variants in exons	Percentage of SNPs/InDels in exons

WS5: SM Comparison

Small Mutation Comparison								
**Name**	**# Overlapping variants**	**# Unique variants**	**% Overlap**	**Total # variants**	**Total % overlap**	**% unique variants**	**# Matching genotype**	**% Matching genotype**
**FCS vs PF-MCB**								
FCS	1740512	978707	64,0	2719219	52,0	29,2	1696249	97,5
PF-MCB		629534	73,4	2370046		18,8		
**PF-MCB vs PF-6months**								
PF-MCB	1688577	681469	71,2	2370046	52,5	21,2	1648888	97,6
PF-6months		843887	66,7	2532464		26,3		
**PF-MCB vs no-Gln**								
PF-MCB	1777770	592276	75,0	2370046	54,3	18,1	1738504	97,8
no-Gln		903193	66,3	2680963		27,6		
**PF-MCB vs K1/1D9-MCB**								
PF-MCB	1733434	636612	73,1	2370046	51,0	18,7	1687702	97,4
1D9-MCB		1029252	62,7	2762686		30,3		
**K1/1D9-MCB vs K1/1D9-3months**								
1D9-MCB	2020797	741889	73,1	2762686	57,6	21,2	1968503	97,4
1D9-3months		743890	73,1	2764687		21,2		

All vs All		
**# Overlapping variants**	**All**	1032151
**% Overlap**	**FCS**	38,0
**% Overlap**	**PF-MCB**	43,5
**% Overlap**	**PF-6mths**	40,8
**% Overlap**	**no-Gln**	38,5
**% Overlap**	**K1/1D9-MCB**	37,4
**% Overlap**	**K1/1D9-3mths**	37,3

Notes

VCFtools vcf-compare v0.1.11 was used to determine the overlapping and unique SMs
SMs = small mutations including SNPs and InDels

Legend

Name: Cell line name
Overlapping variants: number of SMs that were called for both samples
Unique variants: number of SMs that have only been called for one of the samples
% Overlap: percentage of overlapping SMs in relation to the total number of SMs for each sample
# Total variants: total number of SMs for a given cell line
Total % overlap: number of overlapping SMs in the context of all called SMs of the two cell lines compared
% unique variants: percentage of unique variants in the context of all SMs of the two cell lines compared
# Matching Genotype: number of overlapping SMs with a matching genotype
% Matching Genotype: percentage of overlapping SMs with a matching genotype

WS5: SNP Effect

SNP Effect

FCS	
Total SNPs	2340535
Total extension SNPs	1026
Total non-sense SNPs	1081
Total synonymous SNPs	6199
Total intronic/intergenic SNPs	2308851
Total splice-junction SNPs	147
Total mis-sense SNPs	23231
Conservative mis-sense SNPs	6281
Moderately conservative mis-sense SNPs	10599
Moderately radical mis-sense SNPs	4345
Radical mis-sense SNPs	2006

PF-MCB	
Total SNPs	2042781
Total extension SNPs	884
Total non-sense SNPs	924
Total synonymous SNPs	5668
Total intronic/intergenic SNPs	2013887
Total splice-junction SNPs	111
Total mis-sense SNPs	21307
Conservative mis-sense SNPs	5744
Moderately conservative mis-sense SNPs	9642
Moderately radical mis-sense SNPs	4068
Radical mis-sense SNPs	1853

PF-6months	
Total SNPs	2157527
Total extension SNPs	979
Total non-sense SNPs	1020
Total synonymous SNPs	5911
Total intronic/intergenic SNPs	2127503
Total splice-junction SNPs	140
Total mis-sense SNPs	21974
Conservative mis-sense SNPs	5865
Moderately conservative mis-sense SNPs	10051
Moderately radical mis-sense SNPs	4173
Radical mis-sense SNPs	1885

no-Gln	
Total SNPs	2298206
Total extension SNPs	1082
Total non-sense SNPs	1124
Total synonymous SNPs	6367
Total intronic/intergenic SNPs	2265448
Total splice-junction SNPs	139
Total mis-sense SNPs	24046
Conservative mis-sense SNPs	6352
Moderately conservative mis-sense SNPs	10940
Moderately radical mis-sense SNPs	4602
Radical mis-sense SNPs	2152

K1/1D9-MCB	
Total SNPs	2359350
Total extension SNPs	1148
Total non-sense SNPs	1135
Total synonymous SNPs	6965
Total intronic/intergenic SNPs	2323917
Total splice-junction SNPs	144
Total mis-sense SNPs	26041
Conservative mis-sense SNPs	6952
Moderately conservative mis-sense SNPs	11890
Moderately radical mis-sense SNPs	4999
Radical mis-sense SNPs	2200

K1/1D9-3months	
Total SNPs	2366504
Total extension SNPs	1113
Total non-sense SNPs	1142
Total synonymous SNPs	6637
Total intronic/intergenic SNPs	2332536
Total splice-junction SNPs	148
Total mis-sense SNPs	24928
Conservative mis-sense SNPs	6689
Moderately conservative mis-sense SNPs	11407
Moderately radical mis-sense SNPs	4673
Radical mis-sense SNPs	2159

Notes

CooVar v0.07 was used to determine the SNP effect.

Legend

Total SNPs	Total number of SNPs (excluding InDels)
Total extension SNPs	Total number of SNPs eliminating a stop codon
Total non-sense SNPs	Total number of SNPs creating a stop codon
Total synonymous SNPs	Total number of silent SNPs
Total intronic/intergenic SNPs	Total number of SNPs in intronic or intergenic regions
Total splice-junction SNPs	Total number of SNPs in splice junctions
Total mis-sense SNPs	Total number of SNPs causing an amino acid exchange
Conservative mis-sense SNPs	Number of SNPs causing a conservative amino acid exchange
Moderately conservative mis-sense SNP	Number of SNPs causing a moderately conservative amino acid exchange
Moderately radical mis-sense SNPs	Number of SNPs causing a moderately radical amino acid exchange
Radical mis-sense SNPs	Number of SNPs causing a radical amino acid exchange

WS7: SV calls

Normalised SVs

FCS		
**Type**	**# All variants**	**# Filtered variants**
Duplications	2655	591
Deletions	640460	1309
Inversions	1231	284
Translocations	48408	11826
All	692754	14010

PF-MCB-reads		
**Type**	**# All variants**	**# Filtered variants**
Duplications	2726	760
Deletions	717901	2371
Inversions	1258	311
Translocations	47982	12957
All	769867	16399

PF-6months		
**Type**	**# All variants**	**# Filtered variants**
Duplications	3279	787
Deletions	856522	2242
Inversions	1497	377
Translocations	64010	15705
All	925308	19111

no-Gln		
**Type**	**# All variants**	**# Filtered variants**
Duplications	2854	662
Deletions	662111	1775
Inversions	1333	343
Translocations	50947	12879
All	717245	15659

K1/1D9-MCB		
	**# All variants**	**# Filtered variants**
Duplications	2964	721
Deletions	625699	1771
Inversions	1400	354
Translocations	53981	13643
All	684044	16489

K1/1D9-3months		
**Type**	**# All variants**	**# Filtered variants**
Duplications	2871	699
Deletions	657704	1773
Inversions	1383	335
Translocations	52702	13439
All	714660	16246

Notes

Structural variants (SVs) were called with Delly v0.5.9

**Variants are filtered by:**	**Duplications**	**Deletions**	**Inversions**	**Translocations**
**broken pairs**	> 5 broken pairs	> 5 broken pairs	> 5 broken pairs	> 5 broken pairs
**length**	> 300 bp in size	> 300 bp in size	> 300 bp in size	

Legend

# All variants	Total number of structural variants
# Filtered variants	Number of quality filtered variants (see notes)
Type	Type of variant (deletions, duplication, inversion or translocation)

I

WS8: SV Annotation

Annotated normalised SVs					
Name	Total # SVs	# Variants in mRNAs	% Variants in mRNAs	# Variants in exons	% Variants in exons
FCS	14010	4549	32,47	1908	13,62
PF-MCB-reads	16399	5693	34,72	2093	12,76
PF-6months	19111	6710	35,11	2506	13,11
no-Gln	15659	5391	34,43	2165	13,83
K1/1D9-MCB	16489	5756	34,91	2374	14,40
K1/1D9-3months	16246	5494	33,82	2254	13,87

Notes

Structural variants (SVs) were annotated with Delly iover v0.5.9

Legend

Name	Cell line name
Total # SVs	Total number of SVs
# Variants in mRNAs	Number of SVs in mRNAs
% Variants in mRNAs	Percentage of SVs in mRNAs
# Variants in exons	Number of SVs in exons
% Variants in exons	Percentage of SVs in exons

WS9: SV comparison

SV comparison						
**Name**	**# Total variants**	**# Overlapping variants**	**# Unique variants**	**% Overlap**	**Total % Overlap**	**% unique variants**
**FCS vs PF-MCB**						
FCS	14010	6693	7317	47,8	28,3	30,9
PF-MCB-reads	16399	6710	9689	40,9		40,9
**PF-MCB vs PF-6months**						
PF-MCB-reads	16399	7848	8551	47,9	28,4	30,9
PF-6months	19111	7867	11244	41,2		40,7
**PF-MCB vs no-Gln**						
PF-MCB-reads	16399	7312	9087	44,6	29,6	36,7
no-Gln	15659	7338	8321	46,9		33,6
**PF-MCB vs K1/1D9-MCB**						
PF-MCB-reads	16399	7177	9222	43,8	28,0	35,9
1D9-MCB	16489	7193	9296	43,6		36,2
**K1/1D9-MCB vs K1/1D9-3months**						
1D9-MCB	16489	8990	7499	54,5	37,9	31,6
1D9-3months	16246	9001	7245	55,4		30,5

Notes

BEDtools intersect v2.17.0 was used to compare the SVs

Legend

Name: Cell line name

Overlapping variants: number of SVs that could be found in both cell lines compared

Unique variants: number of SVs that have only been called for one of the samples

% Overlap: percentage of overlapping SVs in relation to the total number of SMs for each sample

Total % overlap: number of overlapping SVs in the context of all called SVs of the two cell lines compared

Total % unique: number of unique SVs in the context of all called SVs of the two cell lines compared

WS10: Methylation mapping

Mapping								
**Name**	**Per base coverage w/o 0**	**# Raw reads**	**# Filtered reads**	**# Mapped reads**	**% Mapped reads**	**Mean insert size**	**Mean mapping quality**	**% Genome fraction for 3x coverage**
FCS	7,13	178.104.229	144.449.562	104.506.688	72,30	172,07	31,74	70
PF-MCB-exp	7,86	206.060.245	166.613.563	126.164.551	75,70	154,36	31,74	68
PF-MCB-stat	7,54	204.783.265	165.961.399	124.589.518	75,10	152,92	31,61	65
PF-6months	8,10	196.730.322	159.827.145	116.368.834	72,80	164,21	31,21	74
no-Gln	6,50	154.002.296	126.020.413	92.047.091	73,00	166,40	31,26	67
K1/1D9-MCB	8,18	188.660.390	155.016.427	114.173.440	73,60	163,29	31,78	72
K1/1D9-3months	7,97	187.163.983	153.217.753	113.145.289	73,80	156,08	31,80	71

Notes

Bowtie2 v2.2.2 was used for read alignment.
Numbers given for mapped reads refer to best unique hit alignments.
QualiMap v2.0 was used for mapping statistics.

**Legend**	
Name	Mapping for a given cell line
Per base coverage	Average per base coverage of the mapping excluding 0 values
# Raw reads	Total number of raw reads
# Mapped reads	Total number of mapped reads
# Filtered reads	Total number of filtered reads
% Mapped reads	Percentage of mapped reads
Mean insert size	Mean insert size in bp
Mean mapping quality	Mean mapping quality, phred scaled score
% Genome fraction for 3x cover	Percent of genome with at least 3fold coverage

WS11: Methylation comparison

Differential methylation in structural feature context								
**Name**	**promoter regions**	**transcription start sites**	**introns**	**exons**	**genic**	**intergenic**	**genic + intergenic**	**total differential**
**FCS vs PF-Gln-MCB-exp**								
Hypermethylated in FCS	681	168	7019	1787	6188	7394	13582	15953
Hypermethylated in PF-MCB-exp	69	24	1080	288	907	1464	2371	
**PF-Gln-MCB-exp vs PF-Gln-MCB-stat**								
Hypermethylated in PF-MCB-exp	10	2	18	7	18	39	57	137
Hypermethylated in PF-MCB-stat	9	4	30	9	27	53	80	
**PF-Gln-MCB-exp vs PF-Gln-6months**								
Hypermethylated in PF-MCB-exp	192	57	1126	384	951	1131	2082	2984
Hypermethylated in PF-6months	41	18	451	136	387	515	902	
**PF-Gln-MCB-exp vs no-Gln**								
Hypermethylated in PF-MCB-exp	1146	267	11299	3648	9512	13215	22727	25136
Hypermethylated in no-Gln	70	23	808	179	723	1686	2409	
**PF-Gln-MCB-exp vs K1/1D9-MCB**								
Hypermethylated in PF-MCB-exp	865	269	9758	2519	8548	14869	23417	85240
Hypermethylated in K1/1D9-MCB	2046	584	24821	6134	21740	40083	61823	
**K1/1D9-MCB vs K1/1D9-3months**								
Hypermethylated in K1/1D9-MCB	40	19	390	104	323	457	780	2474
Hypermethylated in K1/1D9-3months	73	27	854	237	750	944	1694	

Notes

R/CRAN package methylKit v0.9.2 and R/Bioconductor package GenomicRanges v1.12.5 were used to identify DMRs and to assign DMRs to structural features.

DMRs are defined as 1 kb non-overlapping windows with a minimal difference in methylation of 25% and a q-value < 0.1.

A DMR may span multiple features (e.g., exon and adjacent intron) increasing the counts of both.
The category 'genic' refers to the number of actual gene annotations overlapping at least one DMR, whereas 'intergenic' is defined as everything not covered by any annotation on either strand.

The genome was split into windows of 1kb and each window tested for differential methylation (minimal coverage of 3, minimal difference in overall methylation 25%, q-value <0.1). Results are sorted according to functional annotation of the respective window. Intergenic are all regions not covered by the other classes.
Regions may be counted multiple times if multiple functional annotations are on the same 1kb window.
For total # of DMRs each window is called only once. TSS = transcription start site.

WS12: Differentially methylated promoters between exponential and stationary culture

Chromosome	gene start/end		gene		DMR start/end					Gene ID
chr1_scaffold_151	96946	99146	H671_1g0	+	98001	99000	*	0.002	25.61	ID=H671_1g0768;protein_id=ERE91674.1;product=protein LCHN-like protein
chr1_scaffold_151	97815	100015	H671_1g0	-	98001	99000	*	0.002	25.61	ID=H671_1g0767_A;protein_id=ERE91672.1;product=wee1-like protein kinase 2
chr2_scaffold_47	9555	11755	H671_2g7	-	9001	10000	*	0	62.165	ID=H671_2g7211;protein_id=ERE82749.1;product=hypothetical protein;codon_start=1;db_xref=GI:537222876
chr5_scaffold_117	340385	342585	H671_5g1	-	340001	341000	*	0.082	36.369	ID=H671_5g13705;protein_id=ERE74099.1;product=A disintegrin and metalloproteinase with thrombospondin motif 10;eC_number=3.4.24.-;codon_start=1;db_xref=GI:537170172,InterPro:IPR000884,InterPro:IPR001590,InterPro:IPR002870,InterPro:IPR010294,InterPro:IPR010909;note=GO_function: GO:0004222 - metalloendopeptidase activity [Evidence IEA]%3B GO_function: GO:0008270 - zinc ion binding [Evidence IEA]%3B GO_function: GO:0008233 - peptidase activity [Evidence IEA]%3B GO_process: GO:0006508 - proteolysis [Evidence IEA]
chr6_scaffold_5	560015	562215	H671_6g1	-	562001	563000	*	0.001	49.935	ID=H671_6g16686;protein_id=ERE70030.1;product=nuclear receptor subfamily 4 group A member 2-like protein;codon_start=1;db_xref=GI:537146567,InterPro:IPR000536,InterPro:IPR001628,InterPro:IPR008946,InterPro:IPR013088;note=GO_function: GO:0003700 - sequence-specific DNA binding transcription factor activity [Evidence IEA]%3B GO_function: GO:0008270 - zinc ion binding [Evidence IEA]%3B GO_function: GO:0043565 - sequence-specific DNA binding [Evidence IEA]%3B GO_function: GO:0003707 - steroid hormone receptor activity [Evidence IEA]%3B GO_process: GO:0043401 - steroid hormone mediated signaling pathway [Evidence IEA]
chr6_scaffold_6	3542366	3544566	H671_6g1	-	3544001	3545000	*	0.093	37.333	ID=H671_6g16871_A;protein_id=ERE69886.1;product=astacin-like metalloendopeptidase;eC_number=3.4.24.21;codon_start=1;db_xref=GI:537146313,InterPro:IPR001506,InterPro:IPR006026;note=GO_function: GO:0008237 - metallopeptidase activity [Evidence IEA]%3B GO_function: GO:0008270 - zinc ion binding [Evidence IEA]%3B GO_function: GO:0004222 - metalloendopeptidase activity [Evidence IEA]%3B GO_process: GO:0006508 - proteolysis [Evidence IEA]
chr6_scaffold_6	3542366	3544566	H671_6g1	-	3544001	3545000	*	0.093	37.333	ID=H671_6g16871_C;protein_id=ERE69888.1;product=astacin-like metalloendopeptidase;eC_number=3.4.24.21;codon_start=1;db_xref=GI:537146315,InterPro:IPR001506,InterPro:IPR006026;note=GO_function: GO:0008237 - metallopeptidase activity [Evidence IEA]%3B GO_function: GO:0008270 - zinc ion binding [Evidence IEA]%3B GO_function: GO:0004222 - metalloendopeptidase activity [Evidence IEA]%3B GO_process: GO:0006508 - proteolysis [Evidence IEA]
chr7_scaffold_60	217706	219906	H671_7g1	+	217001	218000	*	0.016	32.595	ID=H671_7g18514;protein_id=ERE67643.1;product=hypothetical protein
chrx_scaffold_52	163296	165496	H671_xg2	+	164001	165000	*	0	31.273	ID=H671_xg20498;protein_id=ERE65121.1;product=eukaryotic translation initiation factor 2 subunit 3-like protein;codon_start=1;db_xref=GI:537123671,InterPro:IPR000795,InterPro:IPR009001,InterPro:IPR015256;note=GO_function: GO:0005525 - GTP binding [Evidence IEA]
chrx_scaffold_72	506735	508935	H671_xg2	-	507001	508000	*	0	32.016	ID=H671_xg20631;protein_id=ERE63928.1;product=membrane-bound transcription factor site-2 protease;eC_number=3.4.24.85;codon_start=1;db_xref=GI:537120611,InterPro:IPR008915;note=GO_process: GO:0006508 - proteolysis [Evidence IEA]

WS13: Coverage evaluation

Small Mutations

Comparison to each other 13.5x K1/1D9-MCB

Coverage	Total SNPs Subsampled 1	Total SNPs Subsampled 2	Jnique SNPs Subsampled	Jnique SNPs Subsampled	iique SNP % Subsampled	nique SNP % Subsampled	Overlap SNP	verlap SNP % Subsampled	verlap SNP % Subsampled	Total Overlap	unique SMs per sample
13.5_1 vs 13.5_2	2762686	2762612	553388	553314	20,0	20,0	2209298	80,0	80,0	66,6	16,7
13.5_1 vs 13.5_3	2762686	2760712	487454	485480	17,6	17,6	2275232	82,4	82,4	70,0	15,0
13.5_2 vs 13.5_3	2762612	2760712	405707	403807	14,6	14,7	2356905	85,3	85,4	74,4	12,8
									**Average total overlap**	**70,4**	**14,8**

Comparison to each other 20x PF-MCB

Coverage	Total SNPs Subsampled 1	Total SNPs Subsampled 2	Jnique SNPs Subsampled	Jnique SNPs Subsampled	iique SNP % Subsampled	nique SNP % Subsampled	Overlap SNP	verlap SNP % Subsampled	verlap SNP % Subsampled	Total Overlap	unique SMs per sample
20_1 vs 20_2	3305296	3304625	510690	510019	15,5	15,4	2794606	84,5	84,6	73,2	13,4
20_1 vs 20_3	3305296	3305197	199451	199352	6,0	6,0	3105845	94,0	94,0	88,6	5,7
20_2 vs 20_3	3304625	3305197	485726	486298	14,7	14,7	2818899	85,3	85,3	74,4	12,8
									**Average total overlap**	**78,7**	**10,6**

Comparison of K1/1D9-MCB vs. K1/1D9-3mths at 13.5x coverage

Type	# Overlapping variants	# Unique variants	% Overlap	Total # variants	Total % overlap	% unique variants	# Matching genotype	% Matching genotype
K1/1D9-MCB	2020797	741889	73,1	2762686	57,6	21,2	1968503	97,4
K1/1D9-3mths		743890	73,1	2764687		21,2		

Comparison of K1/1D9-MCB vs. K1/1D9-3mths at 21x coverage

Type	# Overlapping variants	# Unique variants	% Overlap	Total variants	Total % overlap	% unique variants	# Matching genotype	% Matching genotype
K1/1D9-MCB	3148251	767807	80,4	3916058	68,9	16,8	3082904	97,9
K1/1D9-3mths		650297	82,9	3798548		14,2		

Structural Variants

Comparison to each other 13.5x K1/1D9-MCB

Coverage	Total SVs Subsampled 1	Total SVs Subsampled 2	Unique SVs Subsampled :	Unique SVs Subsampled :	Unique SV % Subsample	Unique SV % Subsamplec	Overlap SV Subsampled 1	Overlap SV Subsampled 2	Overlap SV % Subsample	Total SV overlap	unique SVs per sample
RN_1 vs RN_2	16489	16643	5736	5893	34,8	35,4	10753	10750	65,2	48,0	26,0
RN_2 vs RN_3	16643	16508	4161	4018	25,0	24,3	12482	12490	75,0	60,4	19,8
RN_1 vs RN_3	16489	16508	4982	4993	30,2	30,2	11507	11515	69,8	53,6	23,2
									**Average total overlap**	**54,0**	**23,0**

Comparison to each other 20x PF-MCB

Coverage	Total SVs Subsampled 1	Total SVs Subsampled 2	Unique SVs Subsampled :	Unique SVs Subsampled :	Unique SV % Subsample	Unique SV % Subsamplec	Overlap SV Subsampled 1	Overlap SV Subsampled 2	Overlap SV % Subsample	Total SV overlap	unique SVs per sample
20_1 vs 20_2	36341	36292	10202	10134	28,1	27,9	26139	26158	71,9	56,2	21,9
20_1 vs 20_3	36341	36355	3153	3163	8,7	8,7	33188	33192	91,3	84,0	8,0
20_2 vs 20_3	36292	36355	9631	9705	26,5	26,7	26661	26650	73,5	58,0	21,0
									**Average total overlap**	**66,1**	**17,0**

Comparison of K1/1D9-MCB vs. K1/1D9-3mths at 13.5x coverage

Type	# Total variants	# Overlapping variants	# Unique variants	% Overlap	Total % Overlap	% unique variants
K1/1D9-MCB	16489	8990	7499	54,5	37,9	31,6
K1/1D9-3mths	16246	9001	7245	55,4		30,5

Comparison of K1/1D9-MCB vs. K1/1D9-3mths at 21x coverage

Type	# Total variants	# Overlapping variants	# Unique variants	% Overlap	Total % Overlap	% unique variants
K1/1D9-MCB	27621	15859	11762	57,4	42,7	31,7
K1/1D9-3mths	25320	15833	9487	62,5		25,6

	1D9-MCB vs 1D9-3mths at 13.5x<br>1D9-MCB vs 3mths at 13.	1D9-MCB vs MCB at 13.5x<br>1D9-MCB vs MCB at 13.5	1D9-MCB vs 1D9-3mths at 21x<br>1D9 MCB vs 3mths at 21:	8mM-MCB vs MCB at 20x<br>8mM-MCB vs MCB at 20x
SMs	21,00	15,00	17,00	11,00
SVs	32,00	23,00	32,00	17,00

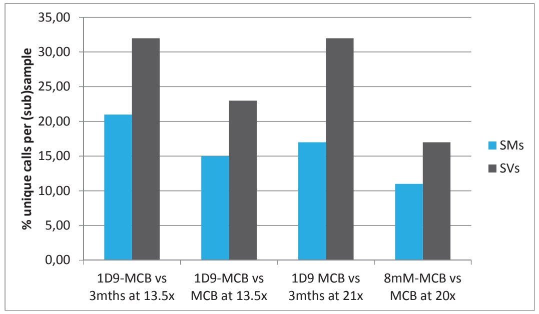

Legend	
Coverage	Coverage
Total SNPs/SVs Subsampl	Total SNPs or SVs found in the subsampled file
Unique SNPs/SVs Subsam	Number of unique SNPs/SVs in the subsampled file
Overlap SNP	Number of overlapping SNPs
Overlap SV Subsampled	Number of overlapping SVs in the subsampled file
Overlap SV Org	Number of overlapping SVs in the original file
Total Overlap	% of overlapping SNPs for all SNPs found in a comparison (shared + unique A + unique B)

WS14: Batch culture sampling points

Batch cultures and sampling points for ChIP-seq and bisulfite sequencing.

Full line: viable cell counts/ml; dashed line: % viability.
Error bars represent values of 8 parallel flasks.
Yellow Arrows depict sampling points for bisulfite sequencing
Blue datapoints: ChIP-seq.

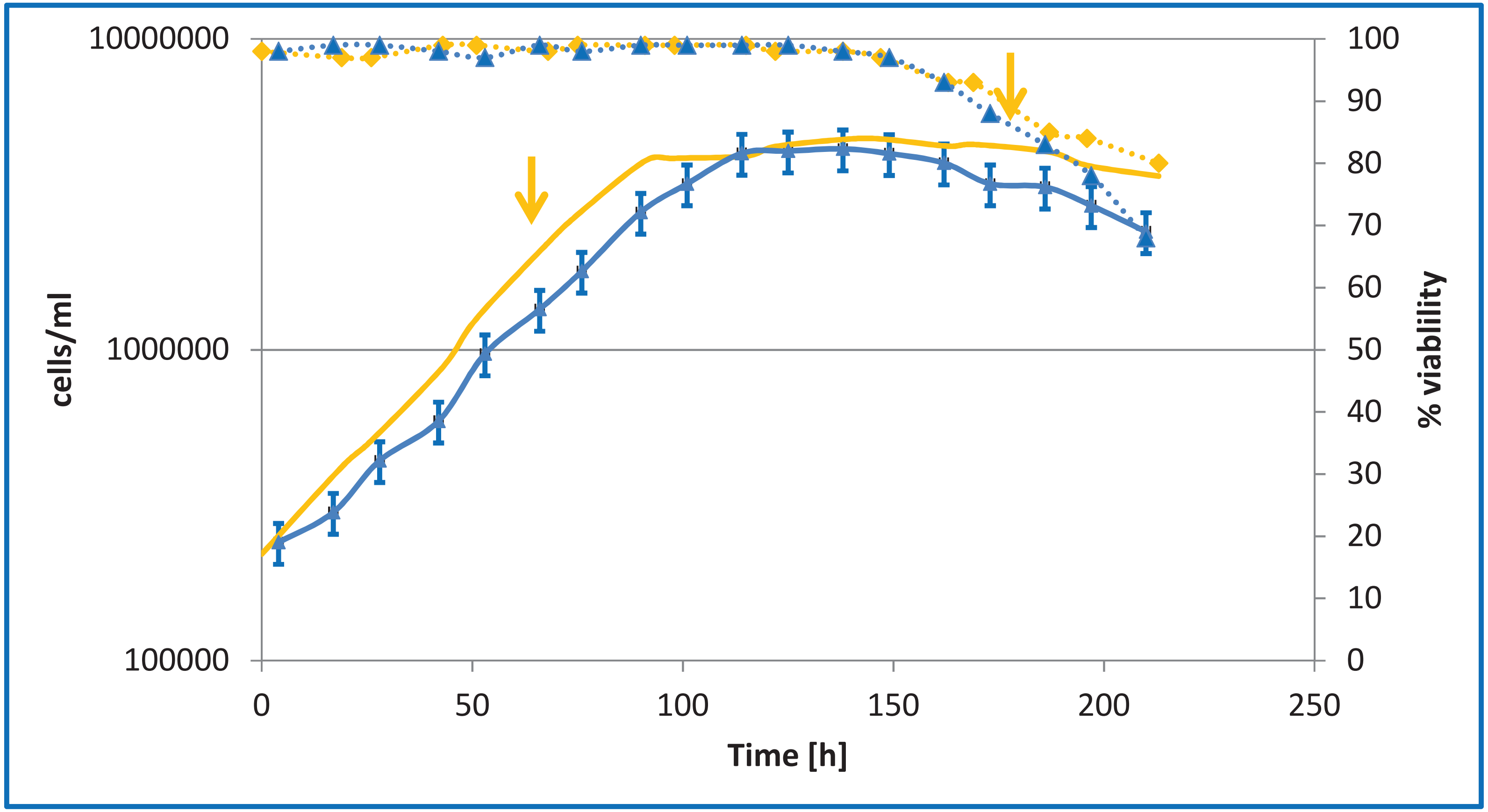

Supplement: Supplementary file 1 — Supporting Information. [file BIT-113-2241-s001.pdf]
